# Prevalence and risk factors of chronic complications among patients with type 2 diabetes mellitus in Morocco: a cross-sectional study

**DOI:** 10.11604/pamj.2022.41.182.25532

**Published:** 2022-03-08

**Authors:** Houda El Alami, Imane Haddou, Ghizlane Benaadi, Mustapha Lkhider, Driss El Habchi, Lahcen Wakrim, Naima Nabih, Omar Abidi, Naima Khlil, Abderrahmane Maaroufi, Abderrahim Naamane, Salsabil Hamdi

**Affiliations:** 1 Environmental Health Laboratory, Institut Pasteur du Maroc, Casablanca, Morocco,; 2 Laboratory of Chemistry, Biochemistry, Nutrition, and Environment, Faculty of Medicine and Pharmacy, University Hassan II, Casablanca, Morocco,; 3 Faculty of Sciences and Techniques of Mohammedia, Morocco,; 4 Institut Pasteur du Maroc, Casablanca, Morocco,; 5 Chrifa Health Center, Casablanca, Morocco,; 6 Higher Institute of Nursing Professions and Health Techniques, Casablanca, Morocco

**Keywords:** Type 2 diabetes mellitus, chronic complications, risk factors, Morocco

## Abstract

**Introduction:**

microvascular and macrovascular complications of type 2 diabetes mellitus (T2DM) are one of the major causes of morbidity and mortality worldwide among patients with T2DM. This study aims to estimate the prevalence of these chronic complications and identify the associated risk factors among Moroccan patients with T2DM.

**Methods:**

this cross-sectional study was conducted on 505 T2DM patients followed by the healthcare Centers of the Casablanca-Settat region from January 2017 to July 2018. The socio-demographic, anthropometric, biochemical, and clinical data were recorded using a structured survey. For statistical analysis, SPSS version 20 is used. Univariate and multivariate logistic regression analyses are used to determine the risk factors associated with chronic complications of T2DM.

**Results:**

among the 505 Moroccan patients with T2DM, 84.98% were women. The average age of the patients was 57.27±10.74 years. Diabetic eye disease was the most frequent complication (29.5%) followed by cardiovascular diseases (CVDs) (22.4%), kidney disease in diabetes (9.8%), diabetes foot (2.8%), and neuropathy (1.8%). Logistic regression analysis showed that the CVDs was associated with hypertension (OR: 2.41; 95% CI: 1.11-5.22; p=0.026), hypolipidemia treatment (OR: 2.20; 95% CI: 1.06-4.59; p=0.034), insulin use (OR= 0.39; 95%CI: 0.15-0.96, p=0.043) and LDL-C (OR: 1.01; 95% CI: 1-1.02; p=0.035) in T2DM patients. However, the major risk factors for the development of kidney disease in T2DM patients were a lack of regular physical activity (OR: 3.77; 95% CI: 1.22–11.67; p=0.021), hypolipidemia treatment (OR: 8.31; 95% CI: 1.86–36.97; p=0.005), and high serum creatinine (OR: 1.33; 95% CI: 1.16-1.53; p≤0.001). In addition, LDL-C levels were found to be a significant risk factor for diabetes eye disease (OR: 1.01; 95% CI: 1.00-1.03; p=0.008).

**Conclusion:**

this study shows that the increased duration of diabetes, insulin use, lack of regular physical exercise, hypertension, hypolipidemia treatment, high serum creatinine, and LDL-C were significant risk factors for chronic complications of T2DM in Moroccan patients.

## Introduction

Diabetes mellitus is frequently reported as a major chronic disease, characterized by abnormal glucose homeostasis leading to hyperglycemia. The prevalence of this disease is increasing dramatically. According to the World Organisation of Health (WHO), the number of people with diabetes worldwide has quadrupled over the past 27 years and reached more than 451 million (8.4%) people in 2017, 91% of whom have type 2 diabetes mellitus (T2DM) [1,2]. This number is predicted to increase to 693 million by 2045 [2]. In Morocco, the prevalence of diabetes is estimated at 12.4% among adults and constitutes a serious public health problem [2]. Regarding the mortality of this disease, 2.2 million deaths worldwide were caused directly by higher blood sugar levels in 2012 and around 1.5 million deaths in 2016, of which more than 80% occurred only in countries of low and middle income [2]. In Morocco, diabetes caused 2224.000 million deaths per year between 2011 and 2015. In addition, the WHO estimates that diabetes will be the 7^th^ leading cause of morbidity and mortality in the world by 2030 [2].

Type 2 diabetes mellitus (non-insulin-dependent diabetes) is a multifactorial disease caused by impaired insulin secretion, abnormalities in its action on target tissues, or a combination of both. However, the pathogenesis of T2DM is not yet fully elucidated and may result from a “gene/gene” or “gene/environment” interaction. On the other hand, environmental factors are well known, related to lifestyles such as sedentary lifestyle, overweight and obesity, unhealthy diet, stress, smoking, intestinal microbiota dysbiosis, and chemical exposure [3-5].

T2DM can cause multiple chronic complications in many parts of the body, such as the eyes, heart, kidneys, nerves, and blood vessels. As a result, it causes several diseases, including hypertension, asthma, cardiovascular diseases (CVDs), blindness, sleep apnea, gynecological problems (menstrual irregularities and infertility), and limb amputations [6-9]. CVDs is more common in patients with T2DM because of the complex interaction of various traditional and nontraditional risk factors that play a principal role in the development and progression of atherosclerosis over its long natural history. These risk factors could include impaired fasting glucose regulation, abdominal obesity, hypertension, and atherogenic dyslipidemia [10,11]. CVDs were found in approximately 32.2% of T2DM patients [12]. Diabetic retinopathy (DR) is a frequent and leading microvascular retinal complication of diabetes. Several studies have shown that DR is the principal cause of visual impairment and blindness in diabetic subjects aged 20 to 65 years, with an estimated global prevalence of 27% during 2015-2019 [13]. The main risk factors for DR are related to age, hyperglycemia, and duration of diabetes. In addition, other factors, including obesity, dyslipidemia, and diabetic nephropathy, were variably associated with DR [14]. Similarly, diabetic nephropathy (DN) is one of the most common microvascular complications of diabetes and the leading cause of end-stage renal disease worldwide [15]. The prevalence of end-stage renal disease in diabetic patients is ten times higher than in non-diabetic patients [16]. T2DM is also associated with an increased risk of premature death, mainly through chronic complications [6]. These problems result in enormous economic costs and pose serious challenges for health policymakers evaluating the current approach to the management of this chronic disease [17].

It is essential to improve prevention and early detection of the disease and develop new, effective therapeutic strategies to address this global problem. This study aims to determine the prevalence and risk factors of micro and macro-vascular complications among subjects with T2DM to better understand the factors contributing to T2DM in Morocco and its complications and improve the overall level of care and management of diabetes with its chronic complications.

## Methods

**Study design:** the data for this cross-sectional study was collected from January 2017 to July 2018 using a detailed survey. We used data collected from T2DM patients aged 20 years or older and treated in health centers in the Casablanca-Settat region of Morocco. The study protocol was approved by the Ethics Committee for Biomedical Research of the Faculty of Medicine and Pharmacy in Rabat. All participants in the study signed a written consent form after receiving all the necessary information regarding the study's objectives.

**Participants and inclusion criteria:** a number of inclusion criteria were used to select the population for this study: 1) Patients with T2DM diagnosed according to international standards; (2) patients older than 20 years; and (3) patients who accept prior informed consent to participate in the study. Exclusion criteria included (1) patients with T1DM or gestational diabetes; (2) patients younger than 20 years; and (3) patients with only one outpatient visit or incomplete data.

**Participants and data collection:** we examined 505 patients with T2DM (75 males and 430 females). The subjects were interviewed face-to-face by trained interviewers using a questionnaire to seize the necessary data. The information collected from the subjects mainly included socio-demographic characteristics (such as age, gender, marital status, education, monthly income, social security, locality type, and ethnicity) and bio-clinical information (such as disease duration, treatment, physical activity, systolic and diastolic blood pressure, waist circumference, BMI, biochemical parameters, and presence of complications). The body mass index was calculated as the ratio of weight (Kg) to height (m) and was classified as underweight (18.5 kg/m^2^), normal-weight (18.5-24.9 kg/m^2^), overweight (25.5-29.9 kg/m^2^), or obese (> 30.0 kg/m^2^) [18]. Blood pressure (systolic and diastolic) was measured according to standard protocols with a validated Microlife BP A100 Plus model. Glycemic control was divided into two categories: HbA1c=7% (good control) and HbA1c >7% (poor control) [19].

**Statistical analysis:** the data was entered into a Microsoft Excel spreadsheet and analyzed with the Statistical Package for Social Sciences (SPSS) version 20.0 for Windows. Continuous data were presented as mean ± standard deviation (SD), while categorical data were presented as frequencies and percentages. The student t-test or Mann-Whitney test were used to compare means for continuous data, whereas associations between different groups and categorical variables were analyzed by the Pearson X^2^ test or Fisher exact test. The univariate logistic regression analysis, followed by the multivariate analysis, was performed to determine the association of independent risk factors with chronic diabetes complications. In the univariable analysis, we selected only significant variables with a p-value <0.05 for inclusion in the multivariable logistic regression model. The independent variables are age, duration of diabetes, physical activity, waist circumference, systolic, blood glucose, serum creatinine, TC, TG, LDL-C, and treatments used for diabetes, diastolic blood pressure, hypertension, and hypolipidemia. On the other hand, the dependent variables are CVDs, renal disease, and diabetic eye disease. Results were expressed as odds ratios (OR) and 95% confidence intervals (CI). A p-value of < 5% is considered statistically significant.

## Results

### Socio-demographic characteristics of patients with T2DM

Among 505 Moroccan T2DM patients who participated in this study, 85,15% were females, and 14,85% were males. The participants´ average age was 57.27 ± 10.74 years and 63.3% were married. More than half of the subjects (58.6%) are illiterate, 70.4% have a monthly income of less than 250 $ and 61.3% have the insurance system (RAMED). Of all the subjects in the study, 91.1% were urban residents, and only 9.1% were smokers or ex-smokers (Table 1). Gender showed a statistically significant relationship between the socio-demographic status indicators that are: age (p=0.053), marital status (p ≤ 0.001), education (p=0.04), monthly income (p≤0.001), and smoking (p ≤ 0.001).

**Table 1:** sociodemographic characteristics of patients with T2DM

Parameter	Total (n=505)	Men (n=75)	Women (n=430)	p-value
**Age (yrs.), Means ± SD**	57.27 ± 10.74	59.35 ± 10.52	56.93 ± 10.75	
20-49	117(23.8%)	12(16.9%)	105(24.9%)	0.053
50-59	162(32.9%)	19(26.8%)	143(34%)	
60 or above	213(43.3%	40(56.3%)	173(41%)	
**Marital Status (%)**				
Single	32(6.4%)	1(1.3%)	31(7.3%)	≤0.001*
Married	317(63.3%)	72(96%)	245(57.5%)	
Widowed	100(20%)	1(1.3%)	99(23.2%)	
Divorced	52(10.4%)	1(1.3%)	51(12%)	
**Education (%)**				
Illiterate	292(58.6%)	30(41.8%)	262(61.6%)	0.04*
Primary	105(21.1%)	18(24.7%)	87(20.5%)	
Secondary	56(10%)	11(15.1%)	39(9.2%)	
Higher Secondary	28(5.6%)	6(8.2%)	22(5.2%)	
Graduate degrees	23(4.6%)	8(11%)	15(3.5%)	
**Monthly income ($) (%)**				
<250	342(70.4%)	35(47.9%)	307(74.3%)	≤ 0.001*
250-800	137(28.2%)	35(47.9%)	102(24.7%)	
>800	7(1.4%)	3(4.9%)	4(1%)	
**Medical insurance (%)**				
Without	93(18.6%)	14(18.7%)	79(18.6%)	0.530
RAMED	306(61.3%)	43(51.3%)	263(62%)	
CNSS	61(12.2%)	9(12%)	52(51.8%)	
Other	39(7.8%)	9(12%)	30(33.1%)	
**Locality type (%)**				
Urban	459(91.1%)	65(86.7%)	394(91.8%)	0.147
Rural	45(8.9%)	10(13.3%)	35(8.2%)	
**Ethnicity (%)**				
Arab	431(87.1%)	68(91.9%)	363(86.2%)	0.147
Berber	42(8.5%)	2(2.7%)	40(9.5%)	
Saharan	22(4.4%)	4(5.4%)	18(4.3%)	
**Smoking (%)**				
Never smoked	459(90.9%)	32(42.7%)	427(99.3%)	**≤** 0.001*
Currently smoking or Ex-smoker	46(9.1%)	43(57.3%)	3(0.7%)	

* p-values were calculated with Chi-square test

### Anthropometric and clinical measurements of patients with T2DM

A large proportion of the diabetic participants (62.5%) had a family history of diabetes, and 37.8% had T2DM for ten years or more. The percentage of consanguineous marriages was higher for males (24,7%) than for females (15%), with a significant statistical difference (p=0.039). Therapeutic modifications, self-monitoring of blood glucose, and medical monitoring were not statistically different between male and female patients (p>0.05). Half of the patients (49.9%) were treated only with oral hypoglycemic agents (OHA), 73.4% of them had HbA1c values > 7%, and 52% were regularly physically active (Table 2). A large majority of the population studied were in the overweight or obesity range (more frequently in women) with 50.4% of cases in obesity (again with significantly higher rates in women; p=0.001). It should be noted that women had a significantly lower waist circumference than men (102.41 ± 12.58 versus 99.97 ± 9.04; p=0.003). Concerning the systolic (S) and diastolic (D) blood pressure (BP), there was not any significant difference between men and women (140.8 ± 19.3/ 76.2 ± 11.8 mm Hg versus 137.6 ± 21.2/76.0 ± 12.2mm Hg; p>0.05). The average serum creatinine levels were significantly higher in males than in females (11.91 ± 4.24 versus 9.61 ± 3.59; p=0.001). In addition, a significant elevation in total cholesterol level (180.32 ± 38.62 versus 195.97 ± 49.6; p=0.036), and LDL-C (104.74± 39.07 versus 117 ± 33.92; p=0.005) were registered. High blood pressure (51.3%) was the most common diabetes-related complication reported from clinical records, followed by dyslipidemia (48.5%), diabetic eye disease (29.5%), cardiovascular diseases (22.4%), diabetic nephropathy (9.8%), diabetic foot (2.8%), and diabetic neuropathy (1.8%) (Table 2).

**Table 2:** clinical and anthropometric measurements of patients with T2DM

Parameter	Total (n=506)	Men (n=75)	Women (n=430)	p-value
**Duration of diabetes (%)**				
<5 years	148(29.4%)	21(28%)	127(29.7%)	0.101
5-10 years	165(32.8%)	18(24%)	147(34.3%)	
>10 years	190(37.8%)	36(48%)	154(36%)	
**Diabetes family history (%)**	308(62.5%)	48(33.3%)	260(61.8%)	0.427
**Consanguinity (%)**	82(16.4%)	18(24.7%)	64(15%)	0.039*
**Therapy modification (%)**	206(44.4%)	25(38.5%)	181(45.4%)	0.299
**Self-monitoring blood glucose (%)**	347(69.1%)	50(68%)	296(69.3%)	0.819
**Medical monitoring (%)**	410(83.3%)	61(81.3%)	349(83.7%)	0.614
**Type of DM treatment (%)**				
Hygienic dietary rules only	46(9.2%)	6(8.1%)	40(9.4%)	0.983
OHA	249(49.9%)	38(51.4%)	211(49.5%)	
Insulin only	122(24.4%)	18(24.3%)	104(24.4%)	
Insulin + OHA	83(16.6%)	12(16.2%)	71(16.7%)	
**HbA1c (%)**				
HbA1c ≤ 7%	120(26.6%)	18(27.7%)	102(26.4%)	0.831
HbA1c > 7%	331(73.4%)	47(72.3%)	284(73.6%)	
**Sedentary (%)**	241(48%)	24(32.4%)	217(50.7%)	0.004*
**BMI in Kg/m2, mean ± SD**	30.64 ± 5.88	26.83±5.67	31.28±5.66	
Underweight	5(1%)	2(2.8%)	3(0.7%)	≤ 0.001*
Normal or healthy weight	51(10.5%)	16(22.5%)	35(8.4%)	
Overweight	185(38.1%)	39(55.9%)	146(35.2%)	
Obese	245(50.4%)	14(19.7%)	231(55.7%)	
**Systolic BP in mmHg, mean ± SD**	13.81± 2.09	14.08 ± 1.93	13.76 ± 2.12	0.124
**Diastolic BP in mmHg, mean ± SD**	7.60 ±1.21	7.62 ± 1.18	7.60 ± 1.22	0.944
**Clinical outcomes, mean± SD**				
HbA1C (%)	8.98 ± 2.45	9.16 ± 2.71	8.95 ± 2.41	0.697
FBG (mg/dl)	199.76 ± 83.23	196.71 ± 70.45	200.3 ± 85.38	0.859
Serum creatinine	9.89 ± 3.75	11.91 ± 4.24	9.61 ± 3.59	≤0.001*
Total cholesterol (mg/dl)	193.97 ± 48.64	180.32 ± 38.62	195.97 ± 49.6	0.038*
Triglyceride (mg/dl)	148.61 ± 90.09	147 ± 101.3	148.8± 88.5	0.462
HDL-C (mg/dl)	52.44 ± 20.61	52.16 ± 19.97	52.48 ± 20.97	0.688
LDL-C (mg/dl)	115.5 ± 34.84	104.74± 39.07	117 ± 33.92	0.005*
**Complications due to DM (%)**				
Hypertension	257(51.3%)	25(33.8%)	232(%)	≤0.001*
Hypolipidemia treatment	243(48.5%)	31(41.9%)	212(49.6%)	0.218
Cardiovascular disease DM	112(22.4%)	14(18.9%)	98(23%)	0.442
Kidney disease in diabetes	49(9.8%)	7(9.5%)	42(9.8%)	0.920
Diabetic eye disease	148(29.5%)	26(35.1%)	122(28.6%)	0.253
Diabetic neuropathy	9(1.8%)	0(0%)	9(2.1%)	0.234
Diabetic foot	14(2.8%)	3(4.1%)	11(2.6%)	0.342

p-values were calculated with the t-test or nonparametric Mann-Whitney U test for quantitative variables or Chi-square test or Fisher's exact tests for categorical ones

### Anthropometric and clinical measurements of patients with T2DM according to the complications of T2DM

According to Table 3, the prevalence of CVDs in diabetic subjects increases significantly with increasing age (p=0.004), increasing duration of diabetes (p=0.001), treatment with a combination of OHAs and insulin (p=0.017), lack of regular physical exercise (p=0.001), hypertension (p=0.001), and hypolipidemia treatment (p=0.001). This prevalence also increases with BMI (p=0.028), waist circumference (p=0.001), systolic BP (p=0.001), total cholesterol (p=0.048), triglycerides (p=0.040), and LDL-C (p=0.002). Results of this study show that kidney disease increases with older age (p=0.010), longer duration of diabetes (p=0.005), therapy with a combination of OHAs and insulin (p=0.006), hypertension (p=0.003), and hypolipidemia treatment (p=0.001). Diabetic patients with a sedentary lifestyle had a higher risk of kidney disease than those who exercise regularly (p=0.016). The higher levels of BMI, serum creatinine, and LDL-C significantly increased the prevalence rates of kidney disease among diabetic subjects (p<0.05). A Comparison of subgroups of diabetic patients with and without eye disease, showed that Increased duration of diabetes (p=0.001), the use of glucose-lowering agents (p=0.004), hypertension (p=0.018), and hypolipidemia treatment (p=0.005) were associated with a significant difference between these two subgroups. The mean total cholesterol levels in diabetic patients with eye disease were significantly higher than in diabetic patients without eye disease (205±47.48 versus 189±48.63; p=0. 010), and it was similar for LDL-C (125±34.88 versus 111±34.12; p=0.001), FBG (213±82.76 versus 193±82.46; p=0.014), and serum creatin (10.58±4.0 versus 9.58±3.58; p=0.020) (Table 3).

**Table 3:** clinical and anthropometric measurements according to the chronic complications of T2DM

Parameter	Cardiovascular diseases			Kidney disease			Diabetic eye disease		
	Yes	No	p-value	Yes	No	p-value	Yes	No	p-value
**Age (%)**									
20-49 years	14.8	26.6	0.004*	18.4	24.6	0.010*	19.9	25.6	0.218
50-59 years	28.7	33.9		18.4	34.4		31.2	33.4	
60 years or above	56.5	39.5		63.3	41		48.9	40.9	
**Duration of diabetes (%)**									
<5 years	18.8	32.7	≤0.001	18.4	30.8	0.005*	18.2	34.4	≤0.001
5-10 years	27.7	34		22.4	33.7		28.4	34.4	
>10 years	53.6	33.2		59.2	35.5		53.4	31.2	
**Type of DM treatment (%)**									
Hygienic dietary rules only	5.4	10.4	0.017*	2	10	0.006*	2	12.3	0.004*
OHA	42.9	51.8		34.7	51.4		51.4	49.1	
Insulin only	26.8	23.6		34.7	23.2		27.7	22.9	
Insulin + OHA	25	14.2		28.6	15.4		18.9	15.7	
**Therapy modification (%)**	50.9	42.5	0.122	50	43.8	0.240	48.6	42.5	0.220
**Medical monitoring (%)**	85.3	83.2	0.590	84.4	83.6	0.878	83.6	83.7	0.976
**Sedentary (%)**	61.6	44.2	≤0.001	64.6	46.3	0.016	52.4	46.3	0.216
**BMI in Kg/m2, mean ± SD**	31.8±5.67	30..3 ± 5.9	0.028	31.1±5.04	30.60±5.96	0.007	31.1±5.78	30.45±5.9	0.155
**Waist circumference (cm)**	106.2±12	100.9±11.9	≤0.001	105±17.7	101±11.4	0.071	102.6±12	101.±12.2	0.493
**SBP in mmHg, mean ± SD**	14.3 ± 1.95	13.65 ± 2.1	≤0.001	14.4 ± 2.28	13.7 ± 2.07	0.086	13.7 ± 1.88	13.8 ± 2.17	0.507
**DBP in mmHg, mean ± SD**	7.72 ± 1.36	7.57 ± 1.17	0.476	7.52 ± 1.30	7.61 ± 1.21	0.667	7.63 ± 1.15	7.59 ± 1.24	0.960
**Hypertension (%)**	75	44.5	≤0.001	71.4	49.1	0.003	59.5	47.9	0.018
**Hypolipidemia treatment (%)**	74.1	41.1	≤0.001	75.5	45.6	≤0.001	58.1	44.5	0.005
**Clinical outcomes**									
HbA1C (%)	9.07 ± 2.41	8.94 ± 2.46	0.513	9.04 ± 2.51	8.96 ± 2.44	0.818	9.06 ± 2.54	8.93 ± 2.40	0.802
FBG (mg/dl)	210 ± 84.7	196± 82.2	0.156	203 ± 83.24	198 ± 83.02	0.669	213 ± 82.76	193 ± 82.46	0.014*
Serum creatinine	10.4 ± 3.97	9.72±3.68	0.109	14.7± 7.09	9.30 ± 2.60	≤0.001	10.58 ± 4.0	9.58 ± 3.58	0.020*
Total cholesterol (mg/dl)	202 ± 54.5	191 ± 46.44	0.048*	188 ± 49.76	194 ± 48.63	0.386	205 ± 47.48	189 ± 48.63	0.010*
Triglyceride (mg/l)	168 ± 109.5	142 ± 82.18	0.040*	169 ± 122.8	146 ± 85.4	0.868	157 ± 94.51	145 ± 88.28	0.256
HDL-C (mg/dl)	50.7 ± 11.7	52.9 ± 23.0	0.768	51 ± 11.76	52.5 ± 21.6	0.970	52 ± 23.31	52.2 ± 19.6	0.709
LDL-C (mg/dl)	127 ± 36.6	111±33.63	0.002*	103.9± 35.1	116± 34.6	0.007*	125 ± 34.88	111 ± 34.12	**≤**0.001

### Risk factors of chronic complication in T2DM

**Cardiovascular diseases:** the univariate logistic regression analysis shows that age (60 years or above OR=2.56; 95%CI: 1.40- 4.70, p=0.002), a long duration of diabetes (>10 years; OR=2.81; 95%CI: 1.61-4.89, p=0.001), the therapy with a combination of OHAs and insulin (OR=3.39; 95%CI: 1.28 -8.96, p=0.014), the hypolipidemia treatment (OR=4.09; 95%CI: 2.56-6.54; p=0.001), lack of regular physical exercise (OR=2.02; 95%CI: 1.31 -3.11; p=0.001), the hypertension (OR=3.74; 95%CI: 2.33-6.0; p=0.001), waist circumference (OR=1.04; 95%CI: 1.02- 1.06, p =0.001), the SBP (OR=1.16 ; 95%CI: 1.05-1.28, p= 0.004), the triglycerides (OR=1.003; 95%CI: 1.0-1.005; p=0.025), and the LDL-Cholesterol level (OR=1.01; 95%CI: 1.0-1.02;p=0.002) are the risk factors of cardiovascular diseases in T2DM (Table 4). In order to identify which of these factors may be related to CVDs of T2DM, multivariate logistic regression analysis was performed. The results show that hypertension (a OR=2.41; 95%CI: 1.11-5.22, p=0.026), hypolipidemia treatment (aOR=2.20; 95%CI: 1.06-4.59, p=0.034), insulin use (aOR=0.39; 95%CI: 0.15-0.96, p=0.043) and LDL-C level (aOR=1.01; 95%CI: 1-1.02, p=0.035) are independent risk factors for CVDs in T2DM in this study (Table 4).

**Table 4:** association of cardiovascular diseases in T2DM with risk factors in logistic regression univariate and multivariate analysis

Parameter	OR (95%CI)	p-value	OR adjusted (95% CI)	p-value
**Age (%)**				
20-49 years	1	-	1	-
50-59 years	1.51(0.78-2.92)	0.214	0.62(0.22-1.74)	0.371
60 years or above	2.56(1.40-4.70)	0.002	0.56(0.25-1.24)	0.158
**Duration of diabetes (%)**				
<5 years				
5-10 years	1.42(0.77-2.60)	0.256	0.55(0.17-1.74)	0.314
>10 years	2.81(1.61-4.89)	≤0.001	0.57(0.26-1.27)	0.176
**Type of DM treatment**				
Hygienic dietary rules only	1	-	1	-
OHA	1.60(0.64-3.99)	0.314	0.77(0.15-3.82)	0.758
Insulin only	2.19(0.84-5.69)	0.105	0.39(0.15-0.96)	0.043
Insulin + OHA	3.39(1.28-8.96)	0.014	0.65(0.26-1.61)	0.359
**Regular exercise**				
Yes (Active)	1	-	1	-
No (Sedentary)	2.02(1.31-3.11)	≤0.001	0.82(0.42-1.63)	0.587
**Waist circumference**	1.04(1.02-1.06)	≤0.001	1.0(0.97-1.03)	0.866
**Systolic BP in mmHg**	1.16(1.05-1.28)	0.004	0.98(0.83-1.17)	0.893
**Hypertension**				
No				
Yes	3.74(2.33-6.0)	≤0.001	2.41(1.11-5.22)	0.026
**Hypolipidemia treatment**				
No	1	-	1	-
Yes	4.09(2.56-6.54)	≤0.001	2.20(1.06-4.59)	0.034
**Clinical outcomes**				
FBG	1.00(0.99-1.00)	0.164	-	-
Serum creatinine	1.04(0.98-1.11)	0.158	-	-
Total cholesterol	1.00(0.99-1.01)	0.083	-	-
Triglyceride	1.00(1.0-1.00)	0.025	0.99(0.99-1.00)	0.730
LDL-C	1.01(1.0-1.02)	0.002	1.01(1.00-1.02)	0.035

**Kidney disease:** the univariate logistic analysis in this study shows that the following risk factors are significantly associated with kidney disease among diabetics: duration of advanced diabetes (>10 years; OR=2.79; 95% CI: 1.28- 6.11, p=0.010), therapy with a combination of OHAs and insulin (OR=9.13; 95% CI: 1.16-71.8; p=0.036), lack of regular physical exercise (OR=2.11; 95% CI: 1.13-3.92; p=0.018), SBP (OR= 1.15; 95% CI: 1.00-1.33; p=0.045), hypertension (OR=2.59; 95% CI: 1.35-4.94; p=0.004), hypolipidemia treatment (OR=3.68; 95% CI: 1.87-7.24, p=0.001), high serum creatinine level (OR=1.32; 95% CI: 1.20-1.45; p=0.001), and LDL-Cholesterol level (OR=0.98; 95% CI: 0.97-1.00; p=0.045) (Table 5). Finally, results of the multivariate logistic regression analysis show that the risk factors for kidney disease among T2DM are as follows: lack of regular physical exercise (aOR=3.77; 95% CI: 1.22-11.67, p=0.021), hypolipidemia treatment (aOR= 8.31; 95% CI: 1.86- 36.97, p=0.005), and high serum creatinine level (aOR=1.33; 95% CI: 1.16-1.53, p=0.001) (Table 5).

**Table 5:** association of kidney disease in T2DM with risk factors in logistic regression univariate and multivariate analysis

Parameter	OR (95% CI)	p-value	OR adjusted (95% CI)	p-value
**Age**				
20-49 years	1	-	-	-
50-59 years	0.71 (0.27-1.86)	0.492	-	-
60 years or above	2.06 (0.94-4.50)	0.068	-	-
**Duration of diabetes**				
<5 years	1			
5-10 years	1.11 (0.45-2.77)	0.811	0.271 (0.04-1.57)	0.146
>10 years	2.79 (1.28-6.11)	0.010	1.252 (0.32-4.88)	0.746
**Type of DM treatment**				
Hygienic dietary rules only	1	-	-	-
OHA	3.31 (0.43-25.5)	0.250	-	-
Insulin only	7.35 (0.95-56.9)	0.056	-	-
Insulin + OHA	9.13 (1.16-71.8)	0.036	-	-
**Regular exercise**				
Yes (Active)	1	-	-	-
No (Sedentary)	2.11 (1.13-3.92)	0.018	3.77 (1.22-11.67)	0.021
**Waist circumference**	1.02 (0.99-1.05)	0.069	-	-
**Systolic BP in mmHg**	1.15 (1.00-1.33)	0.045	0.84 (0.63-1.12)	0.236
**Hypertension**				
No	1	-	-	-
Yes	2.59 (1.35-4.94)	0.004	2.65 (0.8-8.83)	0.111
**Hypolipidemia treatment**				
No	1	-	-	-
Yes	3.68 (1.87-7.24)	≤0.001	8.31 (1.86-36.97)	0.005
**Clinical outcomes**				
FBG	1.00 (0.99-1.00)	0.733	-	-
Serum creatinine	1.32 (1.20-1.45)	≤0.001	1.33 (1.16-1.53)	≤0.001
Total cholesterol	0.99 (0.99-1.00)	0.442	-	-
Triglyceride	1.00 (0.99-1.00)	0.143	-	-
LDL-C	0.98 (0.97-1.00)	0.045	0.98 (0.96-1.00)	0.113

**Diabetic eye disease:** in this study, the univariate logistical regression reveals that the significant risk factors for diabetic eye disease are: long duration of diabetes (OR=3.21; 95% CI: 1.93-5.34; p=0.001), hypertension (OR=1.59 ; 95% CI: 1.08-2.35; p=0.018), FBG level (OR= 1.003; 95% CI: 1.0-1.005; p=0.034), high serum creatinine level (OR= 1.06 ; 95% CI: 1.00-1.13; p=0.030), total cholesterol level (OR= 1.007; 95% CI: 1.0-1.01; p=0.011), LDL-Cholesterol level (OR= 1.01; 95% CI: 1.0- 1.01; p=0.002), hypolipidemia treatment (OR=1.73; 95% CI: 1.17-2.55; p=0.006) and the type of DT2M treatment. Thus, the risk of diabetic eye disease increases by 7.29 times among patients treated with a combination of OHAs and insulin (95% CI: 2.07-25.61; p=0.002), by 7.34 times among patients treated only with insulin (95% CI: 2.14-25.11; p=0.001), and by 6.33 times among patients treated only with OHAs (95% CI: 1.90-21.05; p=0.003) (Table 6). After adjusting for potential confounders, multivariable logistic regression shows that LDL-Cholesterol levels remain significantly different between diabetic subjects with eye disease and diabetic subjects without eye disease (aOR=1.00; 95% CI: 1.00-1.03; p=0.008) (Table 6). In contrast, there was no significant association between eye diseases in subjects with T2DM and the other factors: duration of diabetes, hypertension, hypolipidemia treatment, type of DT2M treatment, FBG level, high serum creatinine level, and total cholesterol level.

**Table 6:** association of diabetic eye disease with risk factors in logistic regression univariate and multivariate analysis

Parameter	OR (95% CI)	p-value	OR adjusted (95% CI)	p-value
**Age**				
20-49 years	1	-	-	-
50-59 years	1.20(0.69-2.08)	0.504	-	-
60 years or above	1.54(0.92-2.58)	0.097	-	-
**Duration of diabetes**				
<5 years	1	-	1	-
5-10 years	1.55(0.90-2.68)	0.112	0.45(0.15-1.35)	0.159
>10 years	3.21(1.93-5.34)	≤0.001	0.59(0.26-1.31)	0.197
**Type of DM treatment**				
Hygienic dietary rules only	1	-	-	-
OHA	6.33(1.90-21.05)	0.003	-	-
Insulin only	7.34(2.14-25.11)	≤0.001	-	-
Insulin + OHA	7.29(2.07-25.61)	0.002	-	-
**Regular exercise**				
Yes (Active)	1	-	-	-
No (Sedentary)	0.78(0.53-1.15)	0.216	-	-
**Waist circumference**	1.00(0.98-1.02)	0.493	-	-
**Systolic BP in mmHg**	0.97(0.88-1.06)	0.530	-	-
**Hypertension**				
No	1	-	1	-
Yes	1.59(1.08-2.35)	0.018	1.55(0.77-3.10)	0.212
**Hypolipidemia treatment**				
No	1	-	1	-
Yes	1.73(1.17-2.55)	0.006	1.26(0.62-2.56)	0.513
**Clinical outcomes**				
FBG	1.0(1.0-1.00)	0.034	1.00(0.99-1.00)	0.117
Serum creatinine	1.06(1.0-1.13)	0.030	1.03(0.95-1.12)	0.406
Total cholesterol	1.00(1.0-1.01)	0.011	0.99(0.98-1.00)	0.262
Triglyceride	1.00(0.99-1.0)	0.271	-	-
LDL-C	1.01(1.0-1.01)	0.002	1.01(1.00-1.03)	0.008

## Discussion

The T2DM micro-macrovascular complications have increased morbidity and mortality significantly worldwide. The importance of our survey is to help determine the prevalence of T2DM complications and their risk factors in the Moroccan population with the aims of facilitating the follow-up of diabetic patients and avoiding the aggravation of the disease. In this cross-sectional study, we included 505 patients with T2DM. The majority of participants were women (85.15%), with a mean age of 57.27±10.74 years. This prevalence is similar to other studies conducted in Morocco. The first one, performed in Fez in 2013, showed that the majority of patients with T2DM were women (77.7%). Similarly, in another study conducted in Oujda in 2011, 64.7% of the diabetic population were women [20,21]. This high percentage of diabetic women in our study can be explained by the fact that we sampled in health centers. The working hours of these proximity centers for routine consultations are mainly in the morning, so most of the patients are housewives.

In this study, 61.3% of the population had the insurance system (RAMED) and 70.4% had a monthly income of less than 250 $. This data explains the access to free care for people in an unfavorable financial situation. Knowing that in Morocco two types of medical coverage have been established: a basic compulsory health insurance (CNOPS for employees in the public sector and CNSS for employees in the private sector) and a medical assistance system (RAMED) based on the principles of social assistance and national solidarity for the benefit of the underprivileged population. Obesity is the leading risk factor for T2DM development. Our study revealed that more than a third of T2DM patients are overweight, and half of this population is obese, with a higher rate in women (55.7%) compared to men (19.7%). These results are similar to those reported in other Moroccan studies [21-24]. This high rate of obesity may be due to the agents used in the treatment of T2DM, including insulin, sulfonylureas, and thiazolidinediones, which promote weight gain [25] Obesity increases the health risks of T2DM and complicates its management. Several studies have shown that patients with T2DM, especially those who are obese, frequently have an atherogenic lipid profile [26]. Indeed, the presence of obesity and T2DM raises the risk of developing hypertension and CVDs [27]. In this regard, a meta-analysis by Zou *et al*. of five articles including 441,199 participants found a significant association between weight fluctuation and an increased risk of CVDs mortality (RR: 1.36, 95% CI 1.22-1.52; p<0.001) and hypertension [28].

The current study also showed that the prevalence of dyslipidemia in diabetics is 48.5%. This result is lower than that reported in Yemen (85%), but higher than that observed in Tunisia (27%) [29]. This variability in the prevalence of dyslipidemia may be due to variations in dietary habits and lifestyle. T2DM is closely associated with an increased risk of CVDs developing, which is further exacerbated by coexisting hypertension. T2DM and high blood pressure are closely associated. In fact, hypertension affects more than half of type 2 diabetic patients [30]. Many physio-pathological mechanisms underlie this association, including inappropriate activation of the renin-angiotensin-aldosterone system (RAAS), oxidative stress, inflammation, impaired insulin-mediated vasodilatation, oxidative stress secondary to excessive production of reactive oxygen species (ROS), and fibrosis, causing microvascular and macrovascular complications of diabetes [30].

The results of our survey revealed that the prevalence of hypertension was 51.3%. Several other studies conducted in different regions of Morocco, between Fes and eastern Morocco, have shown that the prevalence of hypertension ranges between 49.3% and 69.9% in type 2 diabetic patients [20,31,32]. In comparison with other studies conducted in several regions of the world, the prevalence of hypertension in our results was lower than the rates obtained in other studies (64.5% in Qatar [31], 72.4% in Jordan [32], 73% in Spain [33], and 74.4% in Italy [34]). Otherwise, this prevalence is higher than that reported in a study conducted in Tunisia (22%) [29]. Several mechanisms have been proposed to explain this discrepancy between the results obtained and those of other studies, including a higher rate of obesity, genetic predisposition, and environmental factors.

The prevalence of CVDs was about 22.4% in the present study. This result is similar to that conducted in American Indians in Montana (27%) [33] but higher than that obtained in a study conducted in Tunisia (9%) [29] and Saudi Arabia (12.1%) [34]. The variations observed in the prevalence of CVDs among T2DM patients in different countries could be related to differences in the specific characteristics of the population studied, such as late diagnosis of diabetes, late initiation of treatment, or lifestyle factors like unhealthy diet, being overweight, insufficient physical activity, smoking, high blood pressure, and cholesterol [33,34]. Multivariate regression analysis identified hypertension, hypolipidemia treatment, and elevated LDL-Cholesterol as the principal modifiable risk factors for CVDs in patients with T2DM. These results also conform with previous studies from Montana American Indians [33] and Denmark [35]. Prospective studies have also identified the duration of diabetes [34,36] as a risk factor for the development of CVDs in patients with T2DM. However, the present study fails to demonstrate a significant association. Some studies were similar to our results [37]. In the same context, many studies report that cardiac complications development significantly increase mortality in patients with diabetes. This rise varies between 10 and 37% in different studies [38].

The second common complication of T2DM in our samples was eye diseases (29.5%). According to the study by Bos and Agyemang [39], the prevalence of DR shows wide variations between Arab countries, ranging from 8.1% in Tunisia to 41.5% in Egypt. Another cross-sectional study reported that the prevalence of retinopathy in Bangladeshis with T2DM was 12.3% [40]. Many factors can contribute to these variations, including the population, the sample size, and the diagnostic method. Our multivariate regression analysis identified elevated LDL-Cholesterol as an independent risk factor for ocular diseases among T2DM patients. Diabetic nephropathy is a common and morbid complication of diabetes and the leading cause of chronic kidney disease in the developed world. Present study findings show that the prevalence of kidney diseases in our T2DM patients is 9.8%. This prevalence is lower than the rates obtained in other studies (25.2% in Libya, 33.2% in Egypt, 9.2% in Sudan, and 6.3% in Brazil) [41-44]. The observed differences in the prevalence of kidney disease in patients with T2DM in different countries could be since our study was conducted in primary care centers, whereas others were conducted in hospitals that receive more advanced cases addressed to endocrinology and nephrology departments.

On applying multiple regression analysis to diabetes with kidney disease, a positive association was found for a long duration of diabetes (between 5-10 years), lack of regular physical exercise, hypolipidemia treatment, and elevated serum creatinine. These findings were similar to results reported in several other surveys [42,43,45]. The prevalence of diabetic neuropathy (1.8%) and diabetic feet (2.8%) was low in the present study. However, our study results are similar to a study from Eastern Morocco conducted by Sellam *et al*. who reported 3.6% neuropathy and 2% diabetic foot [46]. A study done in Bangladesh reported that the prevalence of neuropathy and diabetic feet was 14.6% and 3.8%, respectively [47]. This low prevalence reported in our study probably reflects the support and education of patients and the long-term follow-up of their disease in the health centers of the Casablanca-Settat region in Morocco.

Chronic complications associated with T2DM alter the health-related quality of life of diabetics, impairing mobility, autonomy, physical activities, and leading to pain, anxiety, and depression. Consequently, they are also responsible for enormous economic and social costs. These chronic complications represent a significant morbidity and mortality burden worldwide.

## Conclusion

This study was mainly based on the studies of the risk factors for chronic complications of T2DM because of their severe consequences. Our findings revealed a very high prevalence of chronic complications of T2DM (cardiovascular diseases, kidney diseases, and eye diseases). Additionally, we have shown that the main risk factors associated with chronic complications of T2DM are, in general, the duration of diabetes, lack of regular exercise, hypertension, hypolipidemia treatment, and LDL cholesterol. However, management of modifiable risk factors can reduce the occurrence of complications and improve care and quality of life for patients. We suggest primary prevention measures such as strict monitoring of blood glucose and blood pressure, physical exercise, and following an appropriate diet. We need a future study to include more subjects with accurate complication status.

### What is known about this topic

- Diabetes prevalence increases suggest an urgent need for action to prevent diabetes and its related complications;
- In Morocco, there is a paucity of research-based that addresses the burden of T2DM-related chronic complications.

### What this study adds

- This paper reports the risk factors associated with complications of T2DM in the Moroccan population;
- This study has shown that diabetic eye disease is the most frequent complication of T2DM, followed by CVDs in Morocco;
- Management of modifiable risk factors can reduce the occurrence of complications.
